# ADA3 regulates normal and tumor mammary epithelial cell proliferation through c-MYC

**DOI:** 10.1186/s13058-016-0770-9

**Published:** 2016-11-16

**Authors:** Nicolas I. Griffin, Gayatri Sharma, Xiangshan Zhao, Sameer Mirza, Shashank Srivastava, Bhavana J. Dave, Mohammed Aleskandarany, Emad Rakha, Shakur Mohibi, Hamid Band, Vimla Band

**Affiliations:** 1 Departments of Genetics, Cell Biology and Anatomy, University of Nebraska Medical Center, 985805 Nebraska Medical Center, Omaha, NE 68198 USA; 2 Departments of Human Genetics Laboratories, Munroe Meyer Institute for Genetics and Rehabilitation, University of Nebraska Medical Center, 985805 Nebraska Medical Center, Omaha, NE 68198 USA; 3 School of Molecular Medical Sciences and Cellular Pathology, University of Nottingham and Nottingham University Hospital, Nottingham City Hospital, Hucknall Road, Nottingham, NG5 1PB USA; 4 Departments of Pathology & Microbiology, College of Medicine, University of Nebraska Medical Center, 985805 Nebraska Medical Center, Omaha, NE 68198 USA; 5 Eppley Institute for Research in Cancer and Allied Diseases, University of Nebraska Medical Center, 985805 Nebraska Medical Center, Omaha, NE 68198 USA; 6 Fred & Pamela Buffett Cancer Center, University of Nebraska Medical Center, 985805 Nebraska Medical Center, Omaha, NE 68198 USA

**Keywords:** ADA3, breast cancer, proliferation, cell cycle, c.-MYC

## Abstract

**Background:**

We have established the critical role of ADA3 as a coactivator of estrogen receptor (ER), as well as its role in cell cycle progression. Furthermore, we showed that ADA3 is predominantly nuclear in mammary epithelium, and in ER+, but is cytoplasmic in ER- breast cancers, the latter correlating with poor survival. However, the role of nuclear ADA3 in human mammary epithelial cells (hMECs), and in ER+ breast cancer cells, as well as the importance of ADA3 expression in relation to patient prognosis and survival in ER+ breast cancer have remained uncharacterized.

**Methods:**

We overexpressed ADA3 in hMECs or in ER+ breast cancer cells and assessed the effect on cell proliferation. The expression of ADA3 was analyzed then correlated with the expression of various prognostic markers, as well as survival of breast cancer patients.

**Results:**

Overexpression of ADA3 in ER- hMECs as well as in ER+ breast cancer cell lines enhanced cell proliferation. These cells showed increased cyclin B and c-MYC, decreased p27 and increased SKP2 levels. This was accompanied by increased mRNA levels of early response genes *c-FOS*, *EGR1*, and *c-MYC*. Analysis of breast cancer tissue specimens showed a significant correlation of ADA3 nuclear expression with c-MYC expression. Furthermore, nuclear ADA3 and c-MYC expression together showed significant correlation with tumor grade, mitosis, pleomorphism, NPI, ER/PR status, Ki67 and p27 expression. Importantly, within ER+ cases, expression of nuclear ADA3 and c-MYC also significantly correlated with Ki67 and p27 expression. Univariate Kaplan Meier analysis of four groups in the whole, as well as the ER+ patients showed that c-MYC and ADA3 combinatorial phenotypes showed significantly different breast cancer specific survival with c-MYC-high and ADA3-Low subgroup had the worst outcome. Using multivariate analyses within the whole cohort and the ER+ subgroups, the significant association of ADA3 and c-MYC expression with patients’ outcome was independent of tumor grade, stage and size, and ER status.

**Conclusion:**

ADA3 overexpression enhances cell proliferation that is associated with increased expression of c-MYC. Expression patterns with respect to ADA3/c-MYC can divide patients into four significantly different subgroups, with c-MYC High and ADA3 Low status independently predicting poor survival in patients.

**Electronic supplementary material:**

The online version of this article (doi:10.1186/s13058-016-0770-9) contains supplementary material, which is available to authorized users.

## Background

Coordination of cell cycle progression with chromosomal duplication maintains genomic stability; a critical cancer-associated trait [1]. Deregulated cell cycle components have now also emerged as key biomarkers and therapeutic targets in cancer [2]. Thus, a better understanding of cell cycle machinery and its aberrations in cancer are of fundamental importance in cell and cancer biology. Recently ADA3, a component of histone acetyltransferase (HAT) complexes, has emerged as a key regulator of cell cycle progression through G1/S and G2/M transitions and in maintaining the genomic stability by governing the faithful segregation of chromosomes [3–5].

Breast cancer is the single most common malignancy in women [6]. Towards identifying novel regulators of cell cycle in hMECs, we previously screened for binding partners of the dominant hMEC-immortalizing oncogene HPV16 E6 and identified ADA3 as an E6-binding protein [7], then showed it’s coactivator function for p53 [7, 8], retinoic acid receptor [9] and estrogen receptors (ER) [10, 11]; other investigators showed its role in androgen receptor [12]. We observed in breast cancer cells that ADA3 was in a large complex that included counterparts of the yeast SAGA complex (Ada2, Ada3 and Gcn5, a HAT), as well as the cell cycle- and cancer-associated HATs, p300 and PCAF [11]. We also demonstrated that ADA3 is essential for p300-mediated p53 acetylation [8]. Together, these studies suggested a potentially important role of ADA3 in breast cancer.

To explore the physiological roles of ADA3, we engineered *Ada3* ^*fl/fl*^ mice and used these to show that germline homozygous deletion of Ada3 was early embryonic lethal [4]. The most dramatic result of conditional deletion of *Ada3* in Ada3^fl/fl^ MEFs was defects in cell cycle progression, including delayed G1 to S transition, mitotic catastrophe, and defective cytokinesis [4], suggesting lack of coordination between DNA replication and subsequent cytokinesis, a precursor for accumulation of DNA damage and genomic instability [13]. Indeed, *Ada3*-null MEFs exhibited increased basal levels of DNA damage response, a delay in the repair of γ-irradiation-induced DNA damage, and increased chromosomal aberrations that increased further upon DNA damage [3], suggesting critical roles of ADA3-dependent histone acetylation in cell cycle-associated transcription, chromatin assembly around newly-synthesized DNA, resolution of stalled replication forks and replication-coupled DNA damage repair [14, 15]. Loss of *Ada3* in MEFs was associated with markedly reduced acetylation of core histones, and reduced levels of p300 and PCAF [4]. Another study using RNAi knockdown showed a role of ADA3 in G2/M progression [16]. Together, these studies demonstrate an essential role of ADA3 in cell cycle progression in MEFs and in tumor cell lines [3, 7–11].

Further studies from our laboratory examined the expression of ADA3 in over 900 breast cancer tissue specimens [17] with known clinico-pathological parameters and survival data. We showed that ADA3 was predominantly nuclear in ER+ breast cancers, consistent with our previous studies that ADA3 functions as an ER coactivator [10, 11], whereas ADA3 expression was both nuclear and cytoplasmic in ER- breast cancers and this expression pattern correlated with high ErbB2/EGFR status and predicted poor patient survival [17].

In this study, we first confirmed our previous studies in *Ada3* ^fl/fl^ MEFs using immortal hMECs where ADA3 depletion led to delayed cell cycle progression with increased p27 and decreased SKP2 levels. Next, we examined the consequence of ADA3 overexpression in immortal hMECs, as well as in ER+ breast cancer cell lines. ADA3 overexpression in both ER- immortal hMECs as well as in ER+ breast cancer cell lines dramatically enhanced cellular proliferation. Cell cycle analyses of ADA3 transfectants showed increased cyclin B, c-MYC and SKP2 levels and decreased p27 levels, findings opposite to those with *ADA3* knockdown. Furthermore, ADA3 overexpression led to increase in mRNA levels of early response genes c-FOS, EGR1 and c-MYC. Analysis of a large cohort of 588 breast cancer tissue specimens showed a significant correlation of ADA3 nuclear expression with c-MYC expression. Furthermore, combinatorial expression of ADA3 and c-MYC showed significant correlation with tumor grade, mitosis, pleomorphism, Nottingham Prognostic Index (NPI), ER/PR status, Ki67 and p27 expression. Multivariate cox regression analysis of the whole cohort or the ER+ subgroups showed significant correlation with tumor grade, stage, and size. Finally, Kaplan Meier analysis showed c-MYC and ADA3 to be independent markers of poor survival as c-MYC high and ADA3 low status predicted poor survival in patients independent of each other.

## Methods

### Cells and Media

76 N-TERT and 81 N-TERT, two immortalized human mammary epithelial cell lines, were grown in DFCI-1 medium, as described earlier [18, 19]. MCF-7 and ZR-75-1 cell lines were grown in α-MEM supplemented with 10% fetal calf serum. For estradiol starvation and stimulation experiments, MCF-7 and ZR-75-1 cell lines were deprived in phenol red-free α-MEM medium (ThermoFisher Scientific, Waltham, MA, USA) supplemented with 5% charcoal stripped fetal calf serum (Atlanta Biologicals, Flowery Branch, GA, USA) and stimulated with 1nM β-estradiol (Sigma, St. Louis, MO, USA) for synchronization experiments [11].

### Antibodies

Generation of anti-ADA3 mouse monoclonal antiserum has been described previously [4]. Antibodies against SKP2 (sc-7164), ERα, Hsc70 (sc-7298), PARP, and β-actin were purchased from Santa Cruz Biotechnology; p27 (610241) and Cyclin B1 (554179) from BD Biosciences; c-MYC (ab32072) from Abcam, Inc; Ki-67 (Clone MIB-1) from Dako. GAPDH (#2118) was obtained from Cell Signaling. H3 (06-755), and H3K56 (07-677) antibodies were from Millipore.

### Generation of Stable Ada3 shRNA Knock-down Cells and ADA3 overexpressing cells

The hAda3-specific RNA sequence used in shRNA constructs is GCAATCAGAACAAGCCCTT and the scrambled shRNA is ACTACGCCTACAGTACGAA [8]. The oligonucleotides were cloned in the pSUPER-Retro vector (OligoEngine, Seattle, WA). 76 N-TERT cells were infected with shRNA retroviral supernatants, as described previously [8]. Virally transduced cells were selected in 0.5 μg/ml puromycin for 3 days, and expression of endogenous ADA3 was assessed in the whole cell lysate using Western blotting with an anti-ADA3 monoclonal antibody [4]. The overexpression construct encoding hADA3 isoform 1 (UniPort KB-075528) was generated by PCR using oligonucleotides and cloning in the pMSCV-Retro vector. 76 N-TERT cells were infected with hADA3 retroviral supernatants, as described above with shRNA infection. Virally transduced cells were selected in 0.5 μg/ml puromycin for 3 days, and expression of endogenous ADA3 was assessed in the whole cell lysate using Western blotting with anti-ADA3 antibody. As an additional approach ADA3 was depleted by using siRNA (sc-7846) and control siRNA (sc-37007) purchased from Santa Cruz Biotechnology, using the same protocol as described in reference [5].

### Cell cycle analysis

76 N-TERT cells expressing scrambled control shRNA or ADA3 shRNA were synchronized in G1 phase of cell cycle using growth factor deprivation by culturing in DFCI-3 medium [18, 19] for 72 hrs, and then released from G1 by switching to growth factor-containing DFCI-1 medium. Cells were collected at 0, 8, 12, 14, 16, 20 and 24-hour time points and then processed for FACS-based cell cycle analysis after propidium iodide staining.

### Proliferation assays

To assess proliferation, cells were plated in 6-well plates in triplicates at a density of 1.0 × 10^4^ cells per well. Trypan blue dye-excluding live Cells were counted on alternate days using a hemocytometer.

### RNA Extraction and Quantitative Real-time PCR

TRIzol reagent (ThermoFisher Scientific, Waltham, MA) was used to isolate total RNA from cells. 2 μg of total RNA was used for reverse transcriptase reaction using SuperScriptTM II reverse transcriptase (Invitrogen). Real-time PCR quantification was performed in triplicates using SYBR Green PCR master mix (Applied Biosystems) and the following primer sets: 1.c-fos - Forward: GGGGCAAGGTGGAACAGTTATC, Reverse: TAGTTGGTCTGTCTCCGCTTGG; 2. EGR1 - Forward: ACCTGACCGCAGAGTCTTTTCC, Reverse: CAGGGAAAAGCGGCCAGTATAG; 3. c-Myc - Forward: TCAAGAGGCGAACACACAAC, Reverse: GGCCTTTTCATTGTTTTCCA; 4. β-actin - Forward: ATCGTCCACCGCAAATGCTTCTA, Reverse: AGCCATGCCAATCTCATCTTGTT The results were calculated by the ΔΔCt method and presented as relative expression after normalization against β-actin.

### Analysis of the p27 Protein Turnover

For analyzing p27 protein half-life in exponentially growing cells, cells were treated with 25 μg/ml of cycloheximide (Sigma, St. Louis, MO) and then harvested at the indicated time points. Total cell extracts were prepared, and equivalent amounts were run on SDS-PAGE and analyzed by Western blotting. The intensity of p27 bands was quantified by densitometry and normalized to β-actin using ImageJ software. Percentage of normalized intensities were calculated and then converted to log values at base 2 and plotted on the Y axis against time of cycloheximide treatment, represented on the X axis [4].

### Nuclear and cytoplasmic fractionation

Nuclear and cytoplasmic fractionation was performed using a commercial kit (ThermoFisher Scientific, Waltham, MA) (78833). Nuclear and cytoplasmic extracts from equivalent numbers of starting cells were run on SDS-PAGE and analyzed by western blotting. PARP and GAPDH respectively were used as controls for nuclear and cytoplasmic markers, respectively to assess the purity of extracts.

### Statistical analysis of ADA3 and c-MYC IHC expression

Immunohistochemistry (IHC) staining of ADA3 in breast cancer tissues was carried out as described previously (nuclear expression of >1% was considered positive) [17]. The high and low nuclear expression of ADA3 was determined as described previously [17]. Nuclear c-MYC staining intensity was similarly determined and presented as MYC-low (negative/weak staining) or MYC-high (moderate/strong staining). Accordingly, the ADA3 and c-MYC co-expression patterns are presented as follows: ADA3Low/ c-MYCLow, ADA3High/c-MYCLow, ADA3Low/ c-MYCHigh, and ADA3High/c-MYCHigh. Associations of these combinatorial patterns with various clinico-pathological as well as molecular markers were determined using the Statistical Package for Social Sciences SPSS version 21 for Windows (Chicago, IL, USA). A p value of less than 0.05 (two-tailed) was considered significant. Cut-off values for the various biomarkers included in this study were chosen before statistical analysis. Standard cut-offs were used for established prognostic factors and were same as previously published for the patient series analyzed here [20]. Analysis of categorical variables was performed with *χ* ^2^ test. Survival curves were generated using the Kaplan–Meier method with significance determined by the Log Rank test. Multivariate analysis was performed using the Cox proportional hazard analysis. A p value (two-sided) < of 0.05 was considered significant.

For IHC analysis of patient derived xenograft (PDX) ER^+^ tumors sections of formalin-fixed and paraffin-embedded tissue samples were obtained from University of Utah and processed for ADA3, ERα and Ki-67 IHC staining, as described previously [17]. These PDX tumors were generated by Dr. Welm’s laboratory by transplanting a portion of a tumor obtained from a patient directly into an immunocompromised mouse [21].

### Karyotype analysis

76 N-TERT vector or ADA3 overexpressing cells were processed for karyotype analysis, as described previously [3].

### Invasion and migration analysis

Cells were deprived of estrogen for 72 hrs by culture in phenol red-free α-MEM medium supplemented with 5% charcoal dextran-stripped fetal calf serum (Atlanta Biologicals, Flowery Branch, GA, USA). The migration assay was done using BD BioCoatTranswell chambers (#354578). 5.0x10^3^ cells in 500 μl of deprivation medium was seeded on top of transwell inserts. Two hours later 700 μl of medium containing 1nM β-estradiol was added to the bottom chamber to serve as the chemoattractant. At the end of the assay, Non-migrated cells on the upper surface of filters were removed by scraping with cheese cloth. The cells at the bottom of inserts were stained using the Hema 3 kit from Fisher (Waltham, WA) and then cells were counted. Invasion assay was performed using BD Matrigel invasion chambers (#354480). Cells were plated, processed and counted similar to migration assay Invasion and migration of ADA3 cells were normalized with respect to vector controls.

### Anchorage-independent growth

2 x10^4^ MCF-7 vector and ADA3 overexpressing cells per well were plated in triplicates in 6-well plates in 2 ml of 0.3% agarose in α-MEM on the top of a bottom layer of 2 ml of 0.6% agarose in α-MEM medium. Cultures were fed every 2 days with 2 ml of α-MEM medium. Twenty-one days after cell seeding, the plates were fixed and stained with 0.05% crystal violet in 25% methanol and colonies in 5 random fields per well were counted.

## Results

### ADA3 is a nuclear protein in ER- immortal hMECs and in ER+ breast cancer cell lines, and is overexpressed in some ER+ breast cancer patient-derived xenografts (PDX)

We have shown that Ada3 deletion in mouse embryonic fibroblasts (MEFs) leads to delay in cell cycle progression. Importantly, cytoplasmic ADA3 expression correlates with poor prognosis and poor survival in ER- breast cancer patients [4, 17]. In this study, we focused on the role of nuclear ADA3 using ER- normal hMECs, ER+ breast cancer cell lines and ER+ primary breast tumor tissues.

**Fig. 1:**
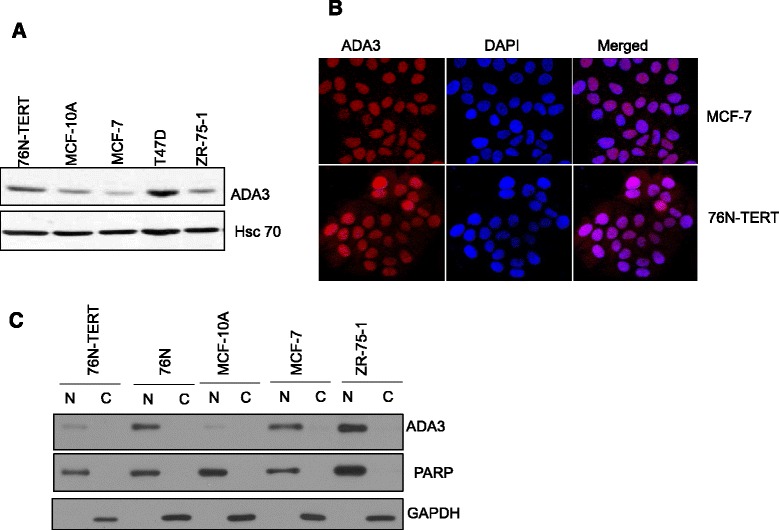
Nuclear localization of ADA3 in immortal and estrogen receptor positive breast cancer cell lines. **a** Western blot analysis of ADA3 expression in immortal hMECs (76 N-TERT, MCF10A) and ER+ breast cancer cell lines (MCF-7, T47D, ZR-75-1). **b** ADA3 immunofluorescence staining in MCF-7 and 76 N-TERT cell lines, DAPI was used for nuclear staining. **c** Nuclear and cytoplasmic fractions were prepared from normal mammary epithelial cell (76 N), immortal mammary epithelial cells (76 N-TERT and MCF10A), and ER+ breast cancer cell lines (MCF-7, ZR-75-1). Protein concentration was quantitated, equal amounts of protein were separated on SDS-PAGE gel and then expression of various protein was assessed with indicated antibodies. PARP was used as a nuclear control, and GAPDH was used as a cytoplasm control

We initially carried out western blotting of total lysates of immortal hMECs and ER+ breast cancer cell lines for ADA3 expression and found all of these cells express ADA3 protein, albeit somewhat different levels (Fig. 1a). Next, we performed immunofluorescence staining with anti-ADA3 antibody as well western blotting of nuclear versus cytoplasmic fractions of these cell lines and found that ADA3 is exclusively localized in the nucleus in both normal hMECs and in ER+ breast cancer cell lines (Fig. 1b, c).

### Overexpression of ADA3 in immortal hMECs enhances proliferation and alters cell cycle regulatory proteins

We previously reported that conditional deletion of Ada3 in *Ada3* ^*fl/fl*^ mouse embryonic fibroblasts (MEFs) leads to defects in cell cycle progression, including delayed G1 to S transition, mitotic catastrophe, and defective cytokinesis [4]. When endogenous ADA3 was depleted in immortal hMECs with retrovirally transduced shRNA, we observed a delay in exit of G1-arrested cells into cell cycle progression together with accumulation of CDK inhibitor p27 and a decrease in the levels of SKP2 (See Additional file 1: Figure S2), supporting a role of ADA3 in hMEC proliferation akin to that in MEFs.

**Fig. 2:**
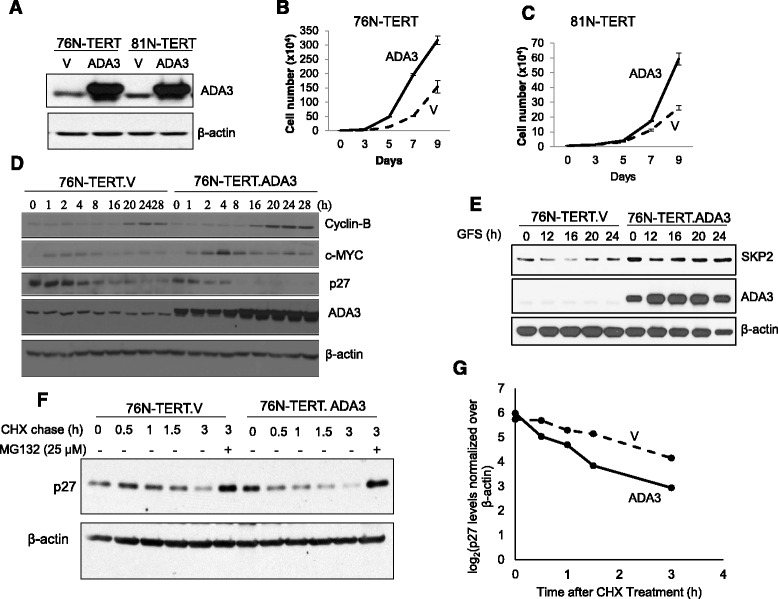
Exogenous overexpression of ADA3 leads to increased proliferation. **a** Western blotting of cells overexpressing ADA3. V = vector. 76 N-TERT cell line (**b**) or 81 N-TERT cell line (**c**) expressing an empty vector (V) or ADA3 were plated at a density of 1 × 10^4^ and then counted using a hemocytometer on alternate days to measure proliferation. **d** Western blotting of cell lysates from synchronized (0 time point) or cells released from synchrony (1-28 hours) were immunoblotted with indicated antibodies. β-actin was used as a loading control. **e** Western blotting of cell lysates from synchronized (0 time point and indicated time points released from synchrony (1-24 hours) were immunoblotted with SKP2, ADA3 or β-actin (used as a loading control). **f** p27 half-life analysis. 76 N-TERT cells expressing vector or overexpressing ADA3 were treated with cycloheximide, and then cells lysates at indicated time points were immunoblotted with anti-p27 antibody. Last lane, 3 hour time point of cells were treated with MG132. **g** The intensity of p27 bands was quantified by densitometry, normalized to β-actin using ImageJ software, and then plotted against the time of cycloheximide treatment

Next, we overexpressed exogenous ADA3 in two ER- immortal hMECs (76 N-TERT and 81 N-TERT) and confirmed ADA3 overexpression using western blotting (Fig. 2a). Immunofluorescence and biochemical fractionation showed that overexpressed ADA3 is found in both nucleus and cytoplasm (Additional file 1: Figure S3) in contrast to exclusive nuclear localization of ADA3 in parental cells.

To assess the impact of overexpressed ADA3 on cell proliferation, we plated equal numbers of vector or ADA3 transduced hMECs (day 0) and then counted the trypan blue-excluding live cells every other day. Notably, ADA3-overexpressing immortal hMECs exhibited a significantly higher rate of proliferation as compared to vector control cells (Fig. 2b, c). However, ADA3 overexpression did not lead to any chromosomal aberrations as assessed by karyotype analyses (Additional file 1: Figure S4).

Given the impact of ADA3 overexpression on cell proliferation, we compared the expression of cell cycle regulatory proteins in vector or ADA3 overexpressing hMECs during cell cycle progression after G1 arrest. Western blotting of lysates at various time periods during cell cycle progression showed that ADA3 overexpressing cells exhibit a more rapid and higher levels of Cyclin B protein accumulation (Fig. 2d). In contrast to results of ADA3 knockdown (Additional file 1: Figure S2B), ADA3 overexpressing hMECs exhibited higher levels of SKP2 and markedly reduced accumulation of p27 during cell cycle progression (Fig. 2d & e). As ADA3 knockout MEFs exhibit a prolonged p27 half-life [4], we assessed the half-life of p27 in vector vs. ADA3 overexpressing hMECs. Cells were treated with cycloheximide to block new protein synthesis (0 time point) and lysates harvested at various time points were western blotted to assess p27 levels. Densitometric quantification showed that the turnover of p27 is substantially faster in ADA3-overexpressing cells compared to that in vector controls (Fig. 2f, g). Treatment with proteasome inhibitor MG132 led to recovery of p27 protein levels in ADA3-overexpressing cells comparable to that in control cells (compare 3 hour lanes with or without MG132) (Fig. 2f, g). The p27 mRNA levels were not affected by ADA3 overexpression (data not shown). Taken together, our results suggest a key role of ADA3 regulation of p27 levels in promoting cell proliferation in hMECs, similar to that seen in MEFs [4].

### Exogenous overexpression of ADA3 in immortal hMECs enhances the induction of early response genes

**Fig. 3:**
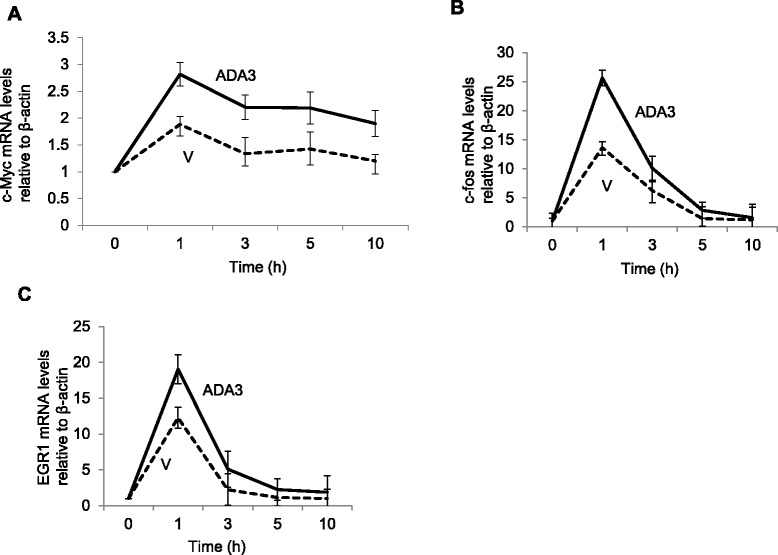
ADA3 transcriptionally regulate early response genes. 76 N-TERT cells vector or ADA3 overexpressing were deprived of growth factors for 72 hours and then released from synchrony via growth factor stimulation. Cells were collected at the indicated time points and total RNA was extracted for real time PCR analyses. Real time PCR analysis of c-myc (**a**), c-fos (**b**) and EGR1 (**c**) is shown

Previous studies using MEFs have shown that ADA3 regulation of SKP2-p27 is mediated by increase in c-MYC levels, we analyzed c-MYC levels in ADA3-overexpressing vs. control hMECs. We observed a significant increase in c-MYC levels in ADA3 overexpressing cells (Fig. 3a). Given the change in c-MYC protein levels, and previous findings that ADA3 as a part of the STAGA complex enhances *c-MYC* transcription [4], we used qPCR to compare the levels of mRNA for *c-MYC* and two other early response genes, *c-FOS* and *EGR1*, in vector vs. ADA3-overexpressing hMECs that were allowed to progress through cell cycle after G1-arrest by growth factor deprivation. The mRNA levels of *c-MYC*, *c-FOS* and *EGR1* increased upon cell cycle exit in both control and ADA3-overexpressing hMECs, peaking at 1 hour; however, ADA3 overexpressing cells expressed higher levels of all three genes as compared to vector control cells, especially at the peak time point (Fig. 3b, c). These results support the idea that ADA3 promotes the expression of *MYC* and other early response genes as part of its ability to promote hMECs proliferation.

### ADA3 overexpression promotes proliferation of ER+ breast cancer cell lines

**Fig. 4:**
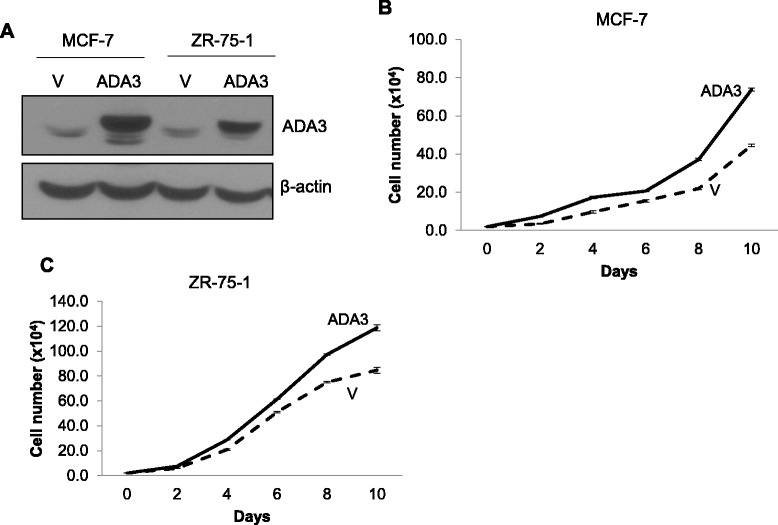
Exogenous overexpression of ADA3 leads to increased proliferation of ER+ breast cancer cell lines. **a** Western blotting of cell lysates from MCF-7 and ZR-75-1 expressing vector or ADA3. MCF-7 (**b**) or ZR-75-1 (**c**) cell lines expressing an empty vector or ADA3 were plated at a density of 1.0x10^4^ cells and then cells were counted on alternate days using a hemocytometer to measure proliferation

Given the results presented above with hMECs, we assessed if ADA3 overexpression in ER+ breast cancer cells also impacts the level of cell proliferation. We obtained vector control or retroviral ADA3 transductants of MCF-7 and ZR-75-1 ER+ breast cancer cell lines both of which express nuclear-localized ADA3 at levels similar to those in immoral hMECs (Fig. 1a). Overexpression was confirmed using western blotting (Fig. 4a). Similar to the results in immortal hMECs (Fig. 2b), ADA3 overexpression in both ER+ breast cancer cell lines led to hyper-proliferation (Fig. 4b, c). As ER+ breast cancer cell lines require estrogen for proliferation [22, 23], we assessed if ADA3 overexpression induced estrogen independent proliferation in MCF-7 or ZR-75-1 cell lines. Comparison of cell proliferation in the absence or presence of estrogen revealed that ADA3 overexpression does not eliminate estrogen dependence (Additional file 1: Figure S5). Taken together, our results demonstrate that ADA3 overexpression promotes hyper-proliferation in both immortal ER- hMECs and ER+ breast cancer cell lines.

### ADA3 overexpression in immortal hMECs or ER+ breast cancer cell lines does not alter cell invasion, migration or anchorage independence

Aside from hyper-proliferation, tumorigenesis requires other traits, such as tumor cells migration and invasion, which may not correlate with the proliferation state [24]. To assess if ADA3 overexpression impacts cell migration or invasion, we compared vector control and ADA3 overexpressing ZR-75-1 cells in standard migration and invasion assays using transwells with or without Matrigel coating, respectively. No significant differences in migration or invasion abilities were observed between vector control and ADA3 overexpressing ZR-75-1 cells (Additional file 1: Figure S6). Furthermore, soft agar colony formation assays showed no differences in the level of anchorage independent growth of control vs. ADA3 overexpressing MCF-7 cells (data not shown). These results support the conclusion that ADA3 overexpression promotes cell proliferation but does not alter cell migration, invasion or anchorage independence of ER+ breast cancer cell lines.

In view of the impact of ADA3 overexpression on ER+ cell proliferation, we examined two available ER+ PDX tumors by IHC to assess ADA3 expression. We observed that one ER+ sample (HCI-003) expressed intermediate levels of ADA3, while the second sample (HCI-011) expressed high levels of ADA3. Concurrent IHC staining for Ki67, a marker of proliferation [25], showed much higher percentage of Ki67+ cells in ADA3 high PDX sample vs. the ADA3 intermediate sample (Additional file 1: Figure S1), prompting further analyses using tissue microarrays derived from a large cohort of ER+ breast cancer patients.

### ADA3 is overexpressed in a subset of ER+ breast cancers, and its overexpression positively correlates with expression of c-MYC

c-MYC plays a key role in breast cancer [26, 27], with gene amplification seen in approximately 15% cases [26] and its overexpression associated with poor outcomes [26, 28, 29]. Given our results in cellular models above, we examined if ADA3 overexpression in ER+ breast cancer patients correlates with c-MYC expression using a large unselected cohort of primary invasive breast cancer specimens that includes various subtypes of breast cancer and in which c-MYC overexpression is known to be significantly associated with poor prognostic factors, including tumor grade and basal-like breast cancer phenotype [30]. c-MYC was also observed to be an independent predictor of a shorter distant metastases-free survival in luminal A LN+ tumors treated with endocrine therapy within this cohort [30]. We have previously assessed the ADA3 expression in this cohort [17]. In this study we used the available ADA3 and c-MYC expression data to assess if ADA3 and MYC expression correlates with each other and whether their relative expression patterns predict outcomes in ER+ breast cancer patients.

**Fig. 5:**
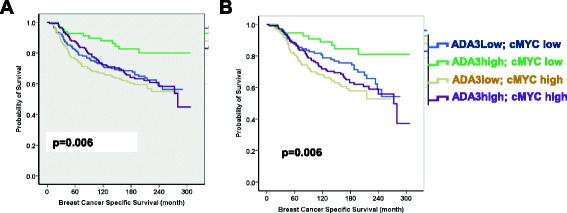
Kaplan Meier plots of nuclear ADA3 and nuclear c-MYC co-expression. Different combinatorial phenotypic groups were categorized; ADA3Low/c-MYCLow; ADA3High/c-MYCLow; ADA3Low/c-MYCHigh; ADA3High/c-MYCHigh as indicated with different colors. **a** in the whole series of breast cancer patients (n = 588 cases) with respect to breast cancer specific survival (BCSS) for 300 months **b**. Kaplan–Meier plot of nuclear ADA3 and nuclear c-MYC co expression combinatorial phenotypic groups within ER + ve tumors only (n = 432 cases) breast cancer patients with respect to BCSS

588 patient samples had informative data on both nuclear ADA3 and c-MYC expression as examined by IHC. Notably, statistically significant positive correlation between c-MYC and ADA3 expression was observed (Table 1). Combinatorial co-expression groups were generated taking nuclear ADA3 and c-MYC expression into account: 172/588 (29.3%) were ADA3Low/c-MYCLow, 69/588 (11.7%) were ADA3High/c-MYC Low, 178/588 (30.3%) were ADA3Low/c-MYCHigh, and 169/588 (28.7%) were ADA3High/c-MYCHigh. Comparing these four groups within the whole unselected series (i.e. ER+ and ER- patient samples) for clinico-pathological parameters, breast cancer molecular subtype, and molecular biomarker expression revealed statistically significant differences between the four groups with respect to tumor grade, mitotic scores, nuclear pleomorphism, tubule formation, Nottingham Prognostic Index (NPI), lympho-vascular invasion (LVI) and histologic tumor types (Table 2). Moreover, statistically significant differences were observed between these groups regarding ER status, PR status, HER2 status, molecular subtype, Ki67 expression and p27 expression (Table 3). Analysis of these groups of patterns of nuclear ADA3 and nuclear c-MYC co-expression within the ER+ tumors only (432 cases) with various clinico-pathological parameters and molecular markers showed a significant association of ADA3/c-MYC co-expression with tumor grade, nuclear pleomorphism, NPI, LVI, histologic tumor type, and the expressions Ki67 and p27 (Table 4). Kaplan Meier survival plots (Fig. 5a and b), (Additional file 1: Table S2 and S3) showed that nuclear ADA3Low/c-MYCHigh or nuclear ADA3High/c-MYCLow status predicted poor outcome in the whole series (A) and in ER+ tumors (B), consistent with our previous analyses that cytoplasmic ADA3 is an independent predictor of poor outcome [17]. Multivariate cox regression analysis for predictors of breast cancer specific survival within the whole cohort and ER+ positive cases showed that ADA3/c-MYC co-expression is significantly associated with patients’ outcome independently of tumor grade, stage, size and ER status (Table 5). Taken together, these results show that i) ADA3 overexpression is seen in a subset of ER+ patients, ii) ADA3 overexpression correlates with c-MYC overexpression, iii) ER+ breast cancers can be categorized into 4 groups ADA3Low/c-MYCLow, ADA3High/c-MYCLow, ADA3Low/c-MYCHigh and ADA3High/c-MYCHigh, iv) these four subgroups showed significant differences in their association with biomarkers and tumors grade and patients’ outcomes, and v) most importantly low nuclear ADA3 or c-MYC High status independently predicts poor survival in ER+ breast cancer patients.

**Table 1:** Significant association between Positive Nuclear expression of ADA3 and c-MYC in unselected breast cancer cases (n = 588 cases)

Parameter	c-MYC Nuclear Expression		Significance	
ADA3 Nuclear Expression	Low No (%)	High No.(%)	*x* ^2^	p value
Low No(%)	172 (49.1)	178 (50.9)	23.79	<0.0001
High No(%)	69 (29.0)	169 (71.3)		

ER+ samples for high and low ADA3 and c-MYC expression showed a statistically significant difference among four groups

**Table 2:** Relationship between nuclear ADA3 and nuclear c-MYC co-expression groups and clinico-pathological parameters within the whole unselected invasive breast cancer series (n = 588 cases)

Parameters		ADA3 Nuclear c-MYC Nuclear expression phenotypes					
		ADA3^low^ c-MYC^low^ N (%)	ADA3^high^ c-MYC^low^ N (%)	ADA3^low^ c-MYC^high^ N (%)	ADA3^high^ c-MYC^high^ N (%)	*X* ^*2*^	P
Patient age	≤50	66 (31.7)	24 (11.5)	65 (31.1)	53 (25.5)	1.862	0.602
	>50	106 (28.0)	45 (11.9)	113 (29.8)	115 (30.3)		
Menopausal Status	Pre	66 (30.4)	25 (11.5)	68 (31.3)	58 (26.7)	0.907	0.871
	Post	106 (28.6)	44 (11.9)	110 (29.7)	110 (29.7)		
Tumor size	≤2 cm	96 (28.0)	14 (12.0)	99 (28.9)	107 (31.2)	2.765	0.429
	>2 cm	76 (31.4)	28 (11.6)	77 (31.8)	61 (25.2)		
Tumor grade	1	20 (23.5)	14 (16.6)	17 (20.0)	34 (40.0)	32.961	**<0.001**
	2	47 (25.3)	18 (9.7)	48 (25.8)	73 (39.2)		
	3	105 (33.3)	37 (11.7)	112 (35.4)	62 (19.6)		
Tubules	1	5 (22.7)	7 (31.8)	5 (22.7)	5 (22.7)	13.345	**0.037**
	2	47 (25.1)	21 (11.2)	59 (31.6)	60 (32.1)		
	3	116 (32.7)	35 (9.9)	107 (30.1)	97 (27.3)		
Pleomorphism	1	1 (14.3)	1 (14.3)	3 (42.9)	2 (28.6)	29.138	**<0.001**
	2	57 (26.0)	31 (14.2)	46 (21.0)	85 (38.8)		
	3	110 (32.6)	31 (9.2)	122 (36.2)	74 (22.0)		
Mitosis	1	43 (24.6)	23 (13.1)	40 (22.9)	69 (39.4)	23.706	**0.001**
	2	30 (29.1)	9 (8.7)	30 (29.1)	34 (33.0)		
	3	95 (33.2)	31 (10.8)	101 (35.3)	59 (20.6)		
Axillary nodal stage	1	93 (28.5)	42 (12.9)	90 (27.6)	101 (31.0)	6.617	0.353
	2	57 (28.2)	23 (11.4)	68 (33.7)	54 (26.7)		
	3	22 (37.3)	4 (6.8)	20 (33.9)	13 (22.0)		
NPI	Good	30 (19.2)	21 (13.5)	39 (4)	66 (25.0)	33.968	**<0.001**
	Moderate	103 (33.0)	40 (12.8)	88 (8.8)	81 (28.2)		
	Poor	39 (33.1)	8 (6.8)	50 (36)	21 (17.8)		
Lymphovascular Invasion	Negative	106 (28.0)	52 (13.8)	103 (27.2)	117 (31.0)	10.624	**0.014**
	Positive	64 (31.4)	16 (7.8)	75 (36.8)	49 (24.0)		
Tumor type	Invasive Ductal/NST	123 (34.6)	39 (11.0)	120 (33.7)	74 (20.8)	61.858	**<0.001**
	Invasive Lobular	13 (22.4)	8 (13.8)	4 (6.9)	33 (56.9)		
	Medullary-like	2 (11.1)	3 (16.7)	9 (50.0)	4 (22.2)		
	Excellent Prognostic Special types*	5 (25.0)	6 (30.0)	5 (25.0)	4 (20.0)		
	Tubular Mixed	20 (20.6)	11 (11.3)	28 (28.9)	38 (39.2)		
	Mixed NST & Lobular	4 (19.0)	2 (9.5)	6 (28.6)	9 (42.9)		
	Mixed NST &other special types	2 (25.0)	0	3 (37.5)	3 (37.5)		

N = number of cases. c. = cytoplasmic, n. = nuclear expression. NST = No Special Type. NPI = Nottingham Prognostic Index

Analysis of all patient specimens from the unselected invasive breast cancer cohort demonstrates a significant correlation between nuclear ADA3 and c-MYC levels in the areas of tumor grade, pleomorphism, tubule formation, mitotic scores, NPI, and tumor type across the four groups

The bold font indicate that the clinical correlation is statistically significant < 0.05

**Table 3:** Relationship between nuclear ADA3 and nuclear c-MYC co-expression combinatorial phenotypic groups with molecular biomarker status within the whole unselected invasive breast cancer series (n = 588 cases)

		ADA3^low^ C MYC^low^ N (%)	ADA3^high^ C MYC^low^ N (%)	ADA3^low^ C MYC^high^ N (%)	ADA3^high^ C MYC^high^ N (%)	Significance	
						*X* ^*2*^	P
ER Status	Negative	59 (39.6)	12 (8.1)	56 (37.6)	22 (14.8)	25.800	<0.001
	Positive	113 (26.0)	56 (12.9)	121 (27.9)	144 (33.2)		
PR Status	Negative	78 (36.0)	25 (10.3)	74 (30.6)	56 (23.1)	12.803	0.005
	Positive	79 (23.7)	43 (12.9)	102 (30.5)	110 (32.9)		
HER2 Status*	Negative	6 (54.5)	2 (18.2)	1 (9.1)	2 (18.2)	21.743	0.010
	Positive	132 (27.0)	55 (11.2)	152 (31.1)	150 (30.7)		
Molecular Subtype	Luminal	99 (25.4)	49 (12.6)	110 (28.3)	131 (33.7)	25.405	<0.001
	HER2 Positive	34 (40.0)	10 (11.8)	24 (28.2)	17 (20.0)		
	Triple Negative	38 (36.2)	7 (6.7)	43 (41.0)	17 (16.2)		
Ki67 labelling** Index	Low	42 (23.0)	27 (14.8)	40 (21.9)	74 (40.4)	22.081	<0.001
	High	83 (29.9)	30 (10.8)	101 (36.3)	64 (23.0)		
p27	Low	114 (38.8)	33(11.2)	89 (30.3)	58 (19.7)	38.276	<0.001
	High	40 (16.7)	33 (13.8)	77(32.1)	90 (37.5)		

*HER2 Status was assessed using American Society of Clinical Oncology/College of American Pathologists Guidelines Recommendations for HER2 Testing in Breast Cancer and Equivocal (2+) HER2+ cases were confirmed by FISH/CISH. ** Ki67 labelling index dichotomized at 14% according to St Galen consensus guidelines 2013

Analysis within the whole unselected invasive breast cancer series with respect to receptor status, molecular subtype, Ki67 labeling index, and p27 demonstrated a statistically significant correlation across the four groups

**Table 4:** Relationship between nuclear ADA3 and nuclear c-MYC co-expression groups with molecular biomarker status within ER+ tumors only (n = 432 cases)

Parameters		ADA3 Nuclear C MYC Nuclear expression phenotypes					
		ADA3^low^ C MYC^low^ N (%)	ADA3^high^ C MYC^low^ N (%)	ADA3^low^ C MYC^high^ N (%)	ADA3^high^ C MYC^high^ N (%)	*X* ^*2*^	P
Patient age	≤50	35 (26.3)	18 (13.5)	39 (29.3)	41(30.8)	0.469	0.936
	>50	78 (26.0)	38 (12.7)	82 (27.3)	102 (34.0)		
Menopausal Status	Pre	35 (24.5)	19(13.3)	44 (30.8)	45 (31.5)	1.002	0.8011
	Post	78 (26.9)	37 (12.8)	77 (26.6)	98 (33.8)		
Tumor size	≤2 cm	65 (24.3)	34 (12.7)	74 (27.6)	95 (35.4)	1.968	0.579
	>2 cm	48(29.3)	22 (13.4)	45 (27.4)	49 (29.9)		
Tumor grade	1	18 (22.0)	14 (17.1)	17 (20.7)	33 (40.2)	15.477	**0.017**
	2	44 (25.6)	16 (9.3)	44 (25.6)	68 (39.5)		
	3	51 (28.5)	26 (14.5)	59 (33.0)	43 (24.0)		
Tubules	1	5 (22.7)	7 (31.8)	5 (22.7)	5 (22.7)	9.813	**0.133**
	2	41 (25.2)	17 (10.4)	51 (31.3)	54 (33.1)		
	3	65 (28.1)	27 (11.7)	60 (26.0)	79 (34.2)		
Pleomorphism	1	1 (14.3)	1 (14.3)	3 (42.9)	2 (28.6)	19.460	**0.003**
	2	52 (24.9)	31 (14.8)	42 (20.1)	84 (40.2)		
	3	58 (29.1)	19 (9.5)	71 (35.7)	51 (25.6)		
Mitosis	1	40 (24.0)	22 (13.2)	38 (22.8)	67 (40.1)	10.175	**0.117**
	2	23 (25.3)	8 (8.8)	30 (33.0)	30 (33.0)		
	3	48 (30.4)	21 (13.3)	48 (30.4)	41 (25.9)		
Axillary nodal stage	1	58 (24.6)	35 (14.8)	61 (25.8)	82 (34.7)	3.652	0.724
	2	43 (26.7	17 (10.6)	50 (31.1)	51 (31.7)		
	3	12 (32.4)	4 (10.8)	10 (27.0)	11 (29.7)		
NPI	Good	27 (18.6)	20 (13.8)	37 (25.5)	61 (42.1)	15.643	**0.016**
	Moderate	63 (29.3)	31(14.4)	56(26.0)	65(30.2)		
	Poor	23 (31.5)	5(6.8)	27 (37.0)	18 (24.7)		
Lymphovascular Invasion (LVI)	Negative	75 (26.2)	44 (15.4)	67(23.4)	100 (35.0)	11.090	**0.011**
	Positive	37 (25.3)	12 (8.2)	54 (37.0)	43 (29.5)		
Tumor type	Invasive Ductal/NST	71 (31.0)	28 (12.2)	71 (31.0)	59 (25.8)	44.333	**<0.001**
	Invasive Lobular	13 (22.4)	8 (13.8)	4 (6.9)	33 (56.9)		
	Medullary-like	0(0)	1(33.3)	2 (66.7)	0 (0)		
	Excellent Prognostic Special types*	5 (26.3)	6 (31.6)	5 (26.3)	3 (15.8)		
	Tubular Mixed	16 (17.4)	11 (12.0)	28 (30.4)	37 (40.2)		
	Mixed NST & Lobular	4 (19.0)	2 (9.5)	6 (28.6)	9 (42.9)		
	Mixed NST &other special types	2 (28.6)	0	3 (42.9)	2 (28.6)		
Ki67 labelling Index	Low	35 (23.0)	25 (14.8)	33 (21.9)	68 (40.4)	11.217	0.011
	High	49 (29.9)	22 (10.8)	38 (36.3)	50 (23.0)		
p27	Low	61 (35.5)	21 (12.2)	47 (27.3)	43 (25.0)	18.993	**<0.001**
	High	37 (16.9)	32 (14.6)	67 (30.6)	83 (37.9)		

N = number of cases. c. = cytoplasmic, n. = nuclear expression. NST = No Special Type. NPI = Nottingham Prognostic Index

Analysis of ER+ patient specimens with respect to tumor grade, nuclear pleomorphism, NPI, LVI, histologic tumor type, Ki67 labeling index, and P27 demonstrated a statistically significant correlation across the four groups

The bold font indicate that the clinical correlation is statistically significant < 0.05

**Table 5:** Multivariate cox regression analysis for predictors of breast cancer specific survival (BCSS) within the whole cohort and the ER+ subgroups

Parameter	In the whole cohort		In ER+ only	
	P value	HR (95% CI)	P value	HR (95% CI)
ADA3 combinatorial expression	0.040	1.06 (1.01–1.16)	0.001	1.12 (1.05- 1.96)
Tumor grade	0.002	1.44 (1.14 – 183)	0.001	1.49 (1.17 -1.90)
Tumor stage	<0.001	2.11 (1.73- 2.58)	<0.001	2.21 (1.72- 2.84)
Tumor size	0.015	1.14 (1.07- 1.91)	0.069	1.38 (0.98 - 1.96)
ER status	0.971	0.10 (0.71- 1.39)	-	-

HR: Hazard Ratio, CI: confidence interval

## Discussion

Precisely regulated cell cycle progression is essential for embryonic development as well as adult tissue homeostasis [31]. Coordination of cell cycle progression with chromosomal duplication maintains genomic stability, a critical cancer-associated trait [1]. Accordingly, deregulated cell cycle components have emerged as key biomarkers and therapeutic targets in cancer [2]. Thus, a better understanding of cell cycle machinery and its aberrations in cancer are of fundamental importance in cell and cancer biology. Here, we show that ADA3 promotes cell proliferation in ER- immortal human mammary epithelial cells and ER+ breast cancer cells involving deregulation of cell cycle associated proteins. Importantly, nuclear ADA3 and c-MYC co-overexpression analyses define ER+ breast cancer subsets in which overexpression of c-MYC or loss of nuclear ADA3 independently predict poor survival.

While previous analyses of ADA3 knockout MEFs have implicated this protein in cell cycle progression [4] and we have reported that ADA3 overexpression and cytoplasmic localization in breast cancer patient specimens specifies poor outcomes [17], the roles of nuclear ADA3 in epithelial cells and the importance of its overexpression in ER+ breast cancer were not examined. In this study, we used immortal (non-tumorigenic) hMECs and ADA3 shRNA/siRNA-mediated knockdown and ADA3 overexpression approaches to establish a clear role of ADA3 in proliferation of ER- mammary epithelial cells. Similar to Ada3 knockout in *Ada3* ^fl/fl^ MEFs [4], knockdown of ADA3 in immortal hMECs led to delay in cell cycle progression as seen by delay in G1 to S phase transition, accumulation of p27 and decrease in SKP2 levels, suggesting that ADA3 operates in cell cycle progression in hMECs and MEFs by the same mechanism [32–34]. Additionally, ADA3 regulates mitosis by its association with CENP-B/centromere and regulates segregation of chromosomes to maintain genomic stability [5]. Recently, we demonstrated that ADA3 is acetylated protein and acetylation of ADA3 by its associated HATs is essential for its key role in cell cycle progression [35].

Given that several cell cycle regulatory proteins are upregulated in cancers to help maintain the higher proliferation rates of cancer cells, we analyzed patient derived xenograft tissue from two ER+ breast cancer patients and noted substantially higher ADA3 levels in one tumor specimen; interestingly, the higher ADA3 expression levels correlated with higher proportion of cells positive for Ki67, a marker of cell proliferation. The suggestion from these experiments that overexpressed ADA3 could drive increased proliferation in breast cancer is supported by our analyses of ADA3 overexpression in immortal hMECs, ER+ breast cancer cell lines and correlative analyses in a large cohorts of ER+ breast cancers specimens.

While ADA3 knockdown/knockout significantly halted cell cycle progression (Additional file 1: Figure S2), overexpression of ADA3 significantly promoted cell proliferation (Fig. 2). Notably, hyper-proliferation of ADA3 overexpressing hMECs involved a more rapid transit into cell cycle that was associated with increased Cyclin B levels and downregulation of p27 protein due to rapid turnover (Fig. 2d). Consistent with these conclusions, increased SKP2 levels were seen in ADA3-overexpressing hMECs (Fig. 2e). As our previous results showed that ADA3 is important in cell cycle associated c-MYC transcription [4], we assessed the *c-MYC* as well as two other early response gene, *c-FOS* and *EGR1*, and found that ADA3 overexpression increased the mRNA levels of all three (Fig. 3). These results are consistent with our previous findings where we defined ADA3-c-MYC-SKP2-p27 pathway for role of ADA3 in cell cycle regulation in MEFs. We and others have previously reported that ADA3, as part of STAGA complex, binds to c-MYC enhancer elements [36, 37]. Notably, another transcriptional coactivator, SRC3 has also been shown to enhance cell proliferation when overexpressed [38]. However, we [11] and others have not observed SRC3 to be present in ADA3-containing protein complexes, suggesting that these co-activators promote cell proliferation through distinct mechanisms. C-FOS is known to be important in promoting cell proliferation and EGR1 is important for cell proliferation and migration [39–41]. Thus, elevated expression levels of these proteins could further contribute to increased proliferation seen in ADA3 overexpressing hMECs.

Given a vast body of literature linking c-MYC expression to positive regulation of cell proliferation [42–44] and our results that ADA3 regulates c-MYC levels (Additional file 1: Figure S2), [3, 4], we assessed the impact of overexpressing ADA3 in ER+ breast cancer cell lines and then analyzed the relationship of nuclear ADA3 and c-MYC expression in ER+ breast cancer specimens. Interestingly, overexpression of ADA3 induced hyperproliferation even in ER+ breast cancer cell lines, suggesting that ADA3 protein overexpression in certain ER+ tumors may increase proliferation index. Analyses of a well characterized cohort of breast cancer tissue samples for nuclear ADA3 and c-MYC expression helped categorize breast cancers into four groups; ADA3 Low/c-MYCLow, ADA3High/c-MYCLow, ADA3Low/c-MYCHigh and ADA3High/c-MYCHigh group of patients. Nuclear ADA3 and c-MYC overexpression was positively correlated (Table 1), underscoring our experimental data in cellular systems that ADA3 regulates c-MYC expression (Fig. 2) [3]. Comparison of combined ADA3/c-MYC expression with clinico-pathological parameters showed a significant correlation of the patterns of their co-expression with tumor grade, pleomorphism, NPI status and tumor types, as well as expression of Ki67 and p27 proteins. Similar results were seen when ADA3/c-MYC combinatorial expression was explored in only the ER+ cases. Univariate and multivariate analyses showed that low nuclear ADA3 staining or high c-MYC expression independently predict poor survival in ER+ breast cancers. While ADA3 overexpression correlated with c-MYC overexpression, survival analyses showed that low ADA3 expression serves as an independent marker for poor survival in ER+ breast cancer patients. The association of low nuclear ADA3 levels with poor survival, while somewhat counterintuitive, is consistent with our previous analyses of this tissue cohort in which cytoplasmic localization of ADA3 was found to be a poor prognostic marker in non-ER+ breast cancers [17], It will therefore be of considerable interest to dissect the molecular pathways by which cytoplasmic ADA3 contributes to poor prognosis and poor survival.

## Conclusions

We demonstrate: i) overexpression of ADA3 enhances cell proliferation in immortal and tumor mammary epithelial cells; ii) increased expression of ADA3 correlates with overexpression of c-MYC; iii) ADA3 and c-MYC expression categorize breast cancers into four groups; ADA3Low/c-MYCLow, ADA3High/c-MYCLow, ADA3Low/c-MYC High and ADA3High/c-MYCHigh; iv) c-MYC High and ADA3 Low status independently predicts poor survival in patients.

## Additional file

Additional file 1: Figure S1. High ADA3 expression in patient derived xenografts correlates with elevated Ki67. Are presentative of two ER+ /PR+ tumor grafts from two different breast cancer patients (HCI-003 and HCI-011). Patient-derived xenografts (as described in DeRose et al. [11]) were sectioned and stained with antibodies indicated (brown staining). Blue staining is hematoxilin. **Figure S2.** Knockdown of ADA3 causes a delay in cell cycle progression in immortal hMECs.76N-TERT cells expressing scrambled shRNAor Ada3 shRNAwere deprived of growth factors in DFCI-3 media for 72hrs, then released from synchrony by adding growth factor containing DFCI-1 medium. Cells were then collected at the indicated time points for various analyses. (A) FACS analysis after propidiumiodide staining. Cell cycle profile (G1/S/G2/M) at selected time points is shown (B) Lysates were immunoblotted with indicated antibodies. β-actin was used as a loading control. (C) Lysates from 76N-TERT transfected with ctrl. or *ADA3* siRNA were immunoblotted with indicated antibodies. **Figure S3.** Exogenous overexpression of ADA3 in Human Mammary Epithelial Cells. Endogenous expression of ADA3 in 76N-TERT cells is only nuclear but after over expression of ADA3, expression is observed in both nucleus and cytoplasm. (A) 76N-TERT.V (i and ii) or 76N-TERT. ADA3 cells (iii and iv) stained for ADA3. (B) ADA3 protein levels after biochemical fractionation, PARP and α-tubulin are used as nuclear and cytoplasmic control, respectively. **Figure S4.** ADA3 over expression does not alter chromosomal stability. **A)** Representative karyotype of 76N-TERT vector cells and **B)** representative karyotype of 76N-TERT ADA3 cells. **Figure S5.** ADA3 overexpression does not induce estrogen independence for proliferation. Indicated cell lines overexpressing vector (V) or ADA3 were deprived of estrogen in phenol red-free alpha-MEM medium supplemented with 5% charcoal dextran stripped fetal calf serum for 72hrs and stimulated with 1nM of β-estradiol. Fresh medium was added on alternate days and total number of cells were determined using a hemocytometer and trypan blue exclusion method. **Figure S6.** ADA3 overexpression has no effect on invasion or migration of cells. ZR-75-1, a ER+ breast cancer cell line expressing vector (V) or ADA3 was examined for changes in invasive **(A)** or migratory **(B)** potential using Boyden chamber method. **Table S1.** Karyotypicanalysis of 76N-TERT vector or ADA3 overexpressing cell lines. **Table S2.** Survival analysis in the whole cohort. **Table S3.** Survival analysis in the ER+ cohort Only. (PDF 575 kb)

## Abbreviations

ADA3: Alteration/Deficiency in Activation 3
ATAC: Ada2a-containing complex
EGR1: early growth response protein 1
ER: estrogen receptor
GCN5: general control non-derepressible 5
H3: histone 3
HAT: histone acetyltransferase
HDAC: histone deacetylase
hMECs: human mammary epithelial cells
HPV: human papilloma virus
MEFs: mouse embryonic fibroblasts
PCAF: p300/CBP-associated factor
STAGA: SPT3/TAF9/GCN5 acetyltransferase complex
