# Supplementary material for: ADA3 regulates normal and tumor mammary epithelial cell proliferation through c-MYC
Source: Breast Cancer Res. 2016 Nov 16;18:113. doi: 10.1186/s13058-016-0770-9 (PMC5112670; doi:10.1186/s13058-016-0770-9)
Supplement: Additional file 1: Figure S1. — High ADA3 expression in patient derived xenografts correlates with elevated Ki67. Are presentative of two ER+ /PR+ tumor grafts from two different breast cancer patients (HCI-003 and HCI-011). Patient-derived xenografts (as described in DeRose et al. [11]) were sectioned and stained with antibodies indicated (brown staining). Blue staining is hematoxilin. Figure S2. Knockdown of ADA3 causes a delay in cell cycle progression in immortal hMECs.76N-TERT cells expressing scrambled shRNAor Ada3 shRNAwere deprived of growth factors in DFCI-3 media for 72hrs, then released from synchrony by adding growth factor containing DFCI-1 medium. Cells were then collected at the indicated time points for various analyses. (A) FACS analysis after propidiumiodide staining. Cell cycle profile (G1/S/G2/M) at selected time points is shown (B) Lysates were immunoblotted with indicated antibodies. β-actin was used as a loading control. (C) Lysates from 76N-TERT transfected with ctrl. or ADA3 siRNA were immunoblotted with indicated antibodies. Figure S3. Exogenous overexpression of ADA3 in Human Mammary Epithelial Cells. Endogenous expression of ADA3 in 76N-TERT cells is only nuclear but after over expression of ADA3, expression is observed in both nucleus and cytoplasm. (A) 76N-TERT.V (i and ii) or 76N-TERT. ADA3 cells (iii and iv) stained for ADA3. (B) ADA3 protein levels after biochemical fractionation, PARP and α-tubulin are used as nuclear and cytoplasmic control, respectively. Figure S4. ADA3 over expression does not alter chromosomal stability. A) Representative karyotype of 76N-TERT vector cells and B) representative karyotype of 76N-TERT ADA3 cells. Figure S5. ADA3 overexpression does not induce estrogen independence for proliferation. Indicated cell lines overexpressing vector (V) or ADA3 were deprived of estrogen in phenol red-free alpha-MEM medium supplemented with 5% charcoal dextran stripped fetal calf serum for 72hrs and stimulated with 1nM of β-estradiol. Fresh medium was ad [file 13058_2016_770_MOESM1_ESM.pdf]

Supplementary Figure 1

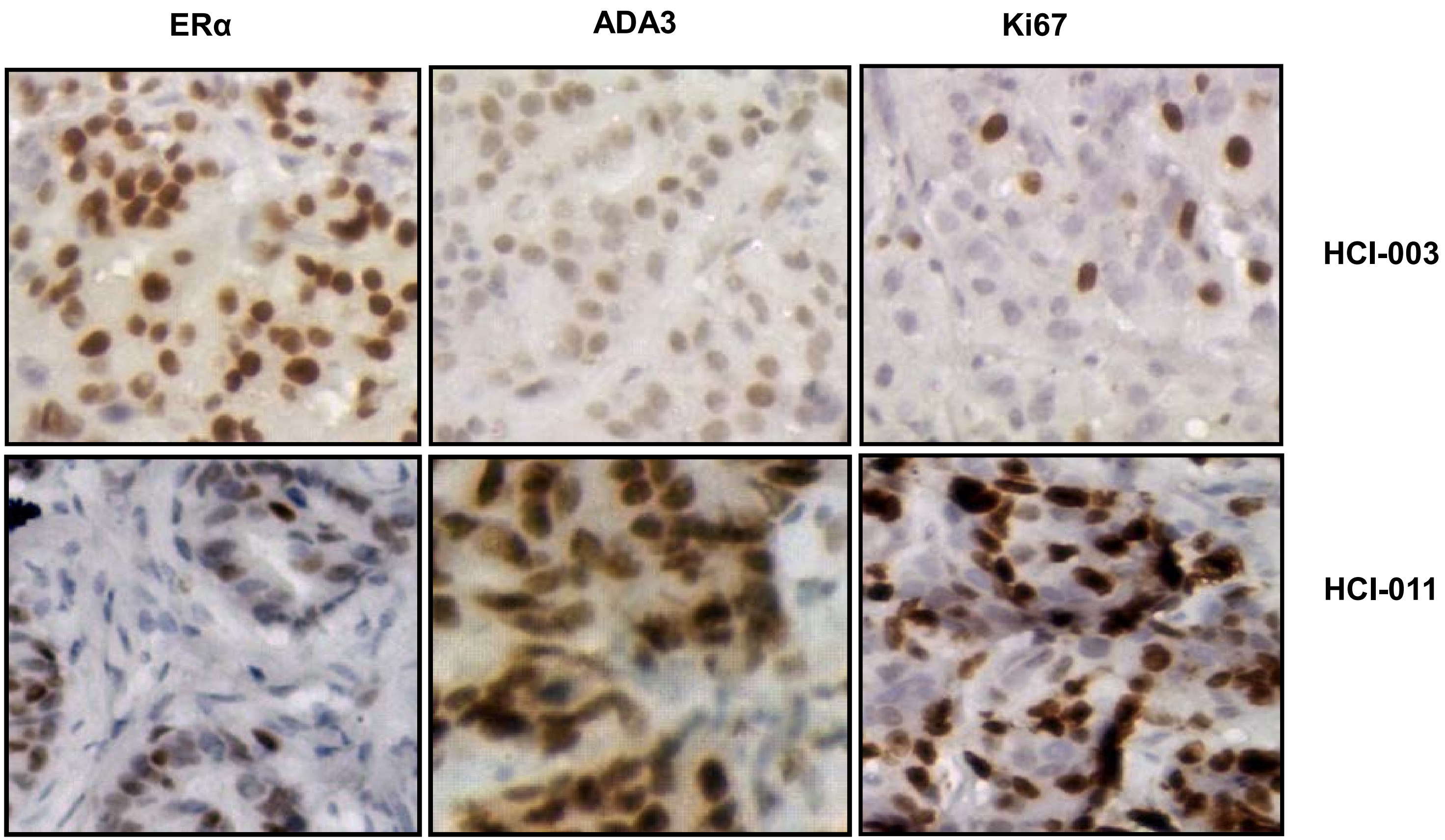

Figure S1. High ADA3 expression in patient derived xenografts correlates with elevated Ki67. A representative of two ER+ /PR+ tumor grafts from two different breast cancer patients (HCI-003 and HCI-011). Patient-derived xenografts (as described in DeRose et al ., 2011) were sectioned and stained with antibodies indicated (brown staining). Blue staining is hematoxilin.

Supplementary Figure 2

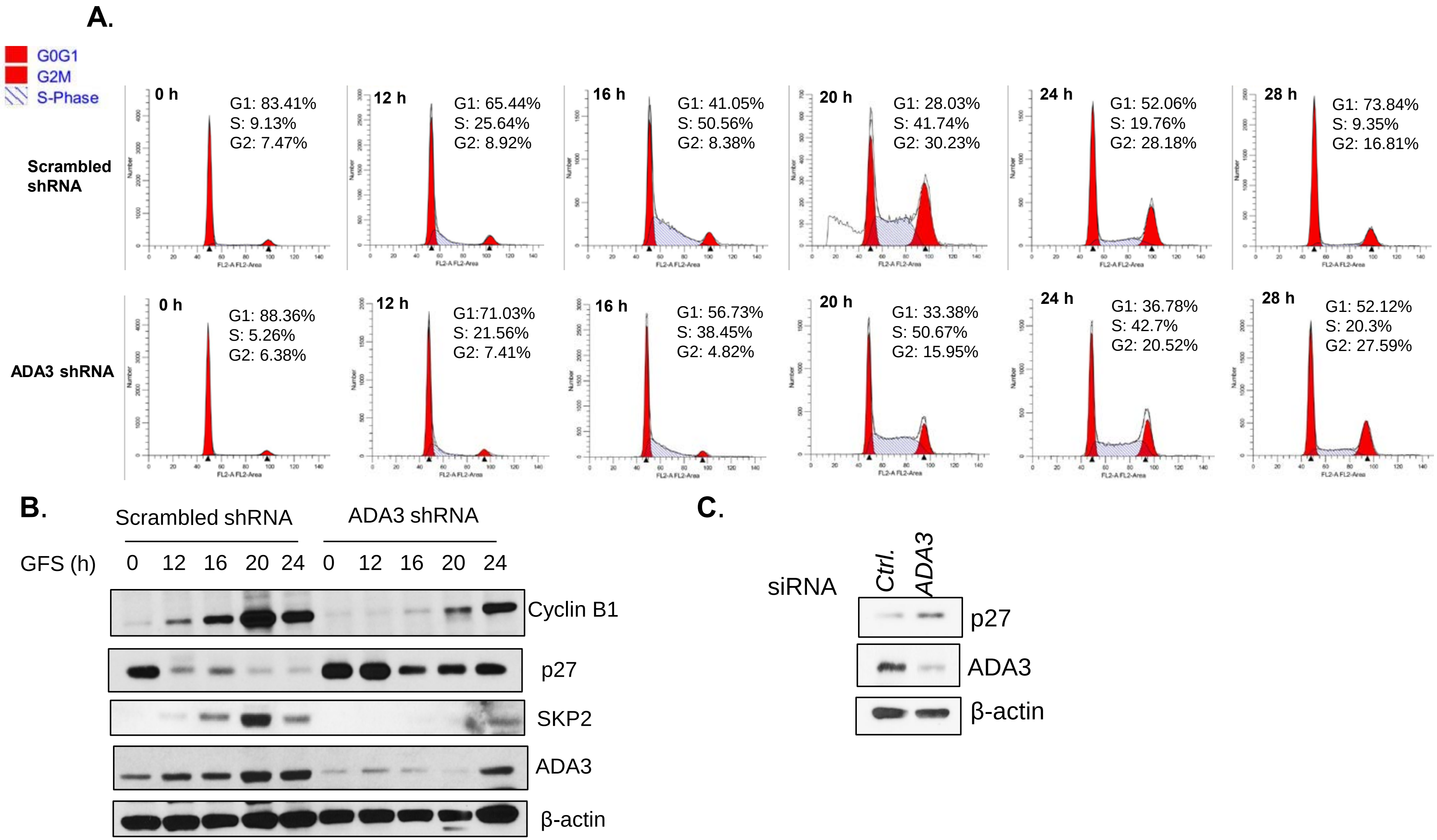

Figure S2. Knockdown of ADA3 causes a delay in cell cycle progression in immortal hMECs. 76N-TERT cells expressing scrambled shRNA or Ada3 shRNA were deprived of growth factors in DFCI-3 media for 72hrs, then released from synchrony by adding growth factor containing DFCI-1 medium. Cells were then collected at the indicated time points for various analyses. **(A)** FACS analysis after propidium iodide staining. Cell cycle profile (G1/S/G2/M) at selected time points is shown **(B)** Lysates were immunoblotted with indicated antibodies. β-actin was used as a loading control. (C) Lysates from 76N-TERT transfected with ctrl. or *ADA3* siRNA were immunoblotted with indicated antibodies.

Supplementary Figure 3

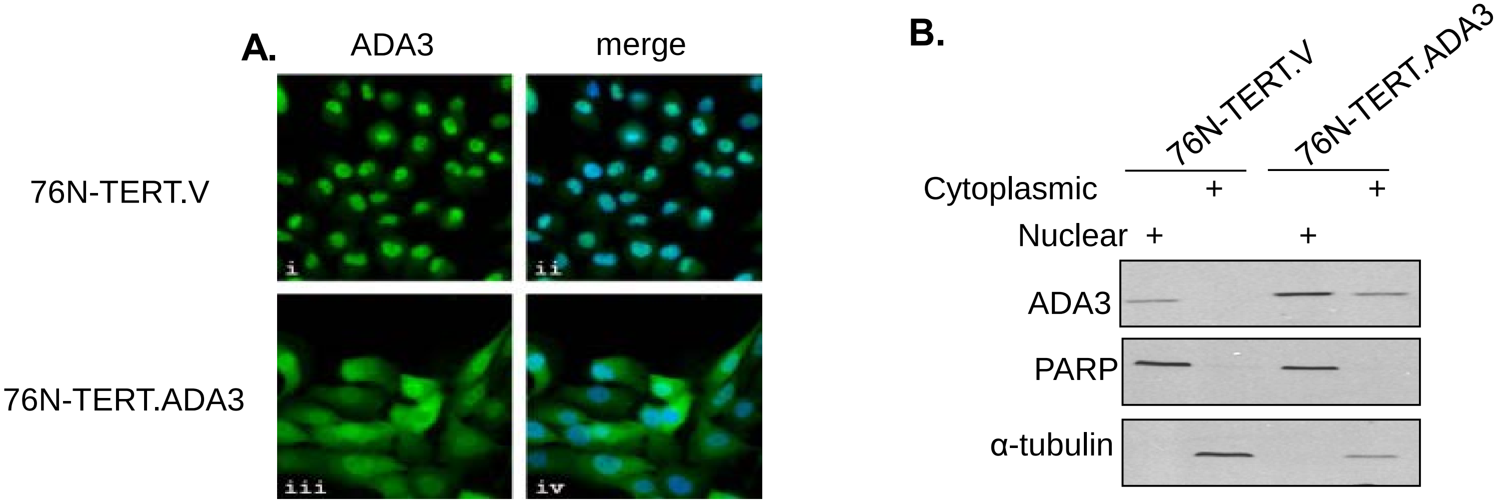

Figure S3: Exogenous overexpression of ADA3 in Human Mammary Epithelial Cells. Endogenous expression of ADA3 in 76N-TERT cells is only nuclear but after overexpression of ADA3, expression is observed in both nucleus and cytoplasm. **(A)** 76N-TERT.V (i and ii) or 76N-TERT.ADA3 cells (iii and iv) stained for ADA3. **(B)** ADA3 protein levels after biochemical fractionation, PARP and α-tubulin are used as nuclear and cytoplasmic control, respectively.

Supplementary Figure 4

A.

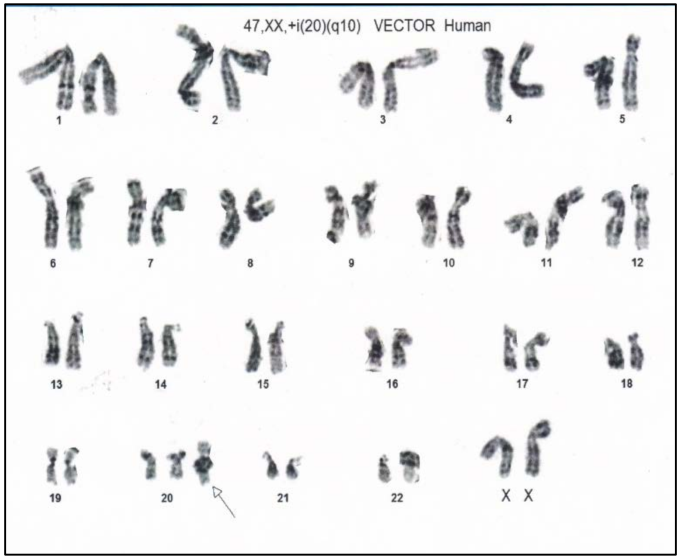

B.

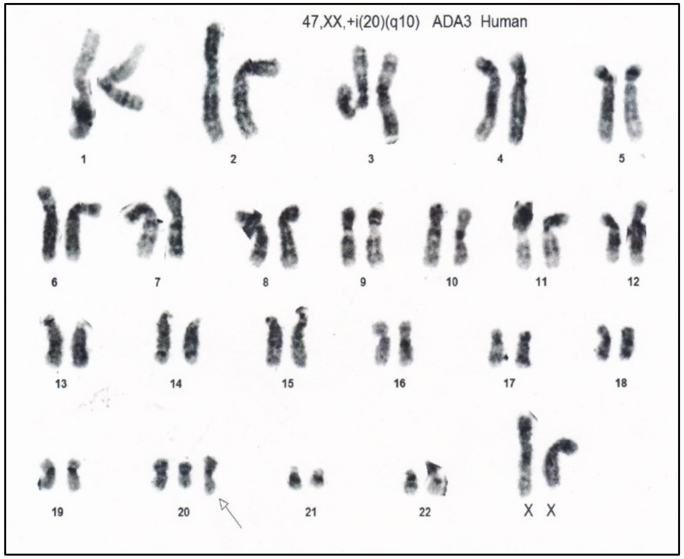

Figure S4: ADA3 overexpression does not alter chromosomal stability. A) Representative karyotype of 76N-TERT vector cells and **B)** representative karyotype of 76N-TERT ADA3 cells.

Table S1. Karyotypic analysis of 76N-TERT vector or ADA3 overexpressing cell lines.

76N-TERT	Total Breaks (in 50 diploid metaphases)	No. of Metaphases scored	Average No. of breaks per metaphase
Vector	19	50	0.38
ADA3	16	50	0.32

Supplementary Figure 5

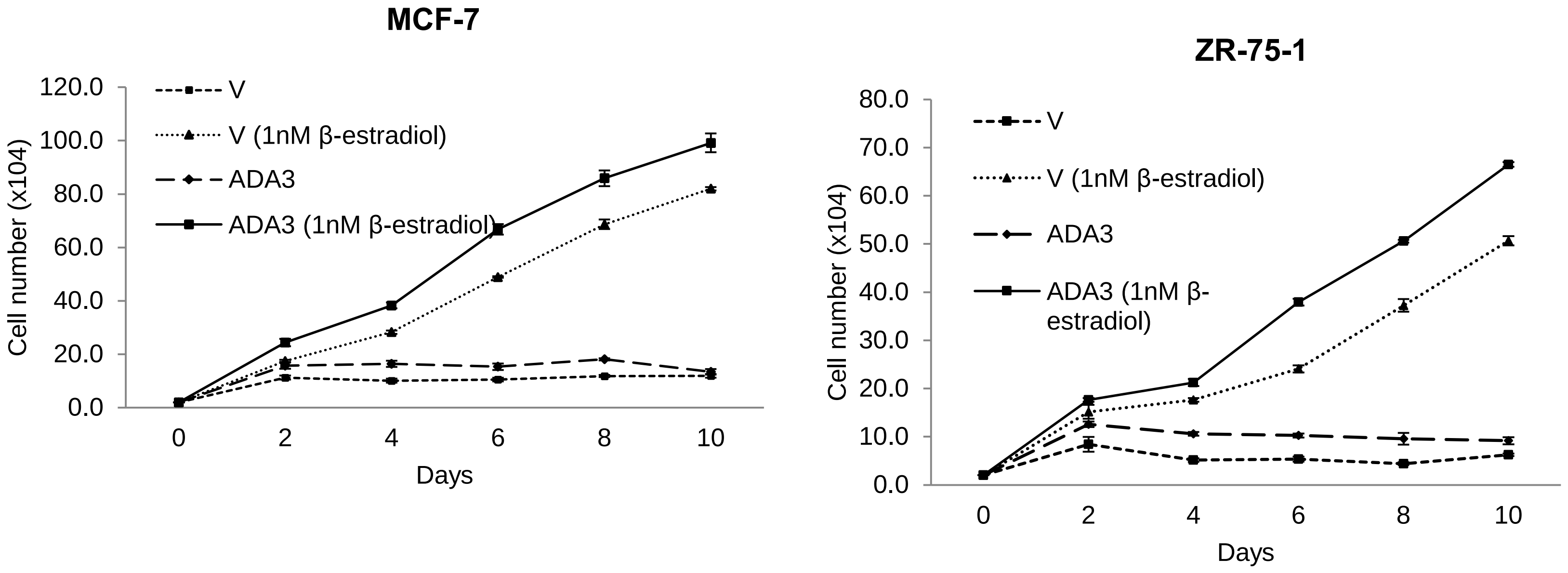

Figure S5. ADA3 overexpression does not induce estrogen independence for proliferation. Indicated cell lines overexpressing vector (V) or ADA3 were deprived of estrogen in phenol red-free alpha-MEM medium supplemented with 5% charcoal dextran stripped fetal calf serum for 72hrs and stimulated with 1nM of β-estradiol. Fresh medium was added on alternate days and total number of cells were determined using a hemocytometer and trypan blue exclusion method.

Supplementary Figure 6

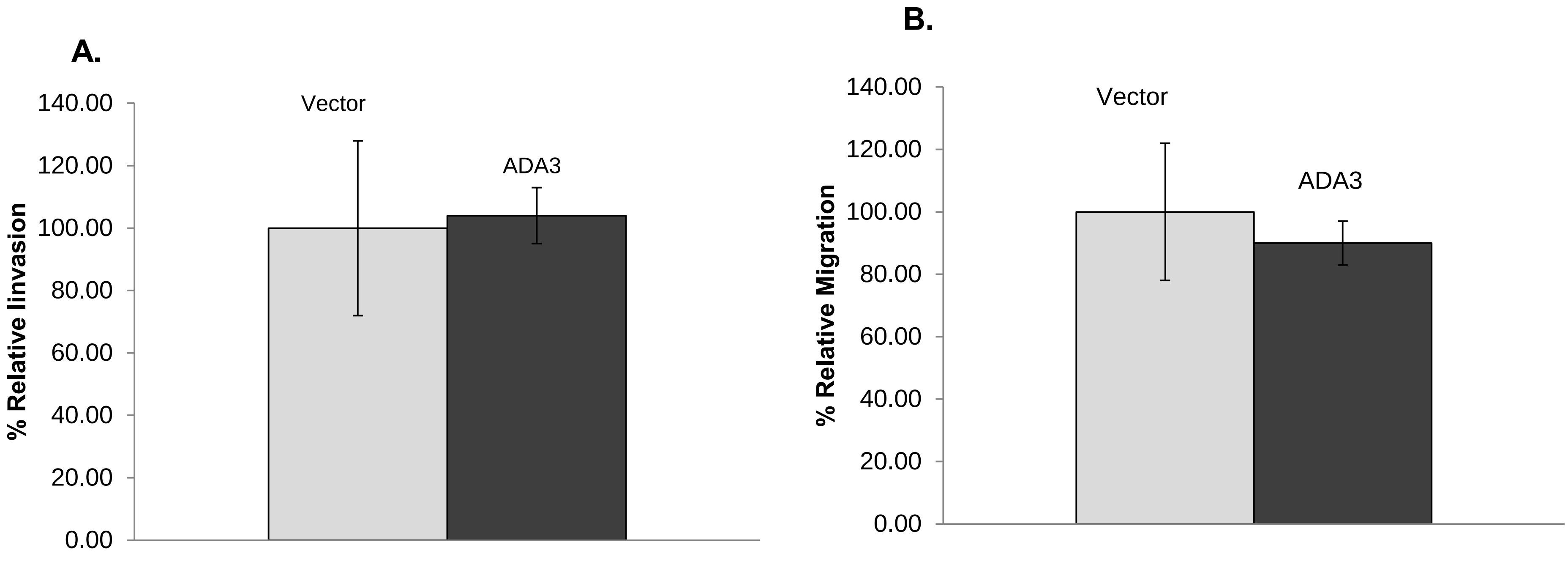

Figure S6. ADA3 overexpression has no effect on invasion or migration of cells. ZR-75-1 , a ER+ breast cancer cell line expressing vector (V) or ADA3 was examined for changes in invasive **(A)** or migratory **(B)** potential using Boyden chamber method.

Table S2:Survival analysis in the whole cohort

Case Processing Summary				
ADA3_NUC_CMYC_COEXPRESSION	Total N	N of Events	Censored	
			N	Percent
ADA3 Negative C MYC Negative	172	60	112	65.1%
ADA3 Positive C MYC Negative	68	11	57	83.8%
ADA3 Negative C MYC Positive	177	70	107	60.5%
ADA3 Positive C MYC Positive	167	60	107	64.1%
Overall	584	201	383	65.6%

Table S3 Survival analysis in the ER+ cohort Only :

ADA3_NUC_CMYC_COEXPRESSION	Total N	N of Events	Censored	
			N	Percent
ADA3 Negative C MYC Negative	113	34	79	69.9%
ADA3 Postive C MYC Negative	56	9	47	83.9%
ADA3 Negative C MYC Positive	120	48	72	60.0%
ADA3 Positive C MYC Positive	142	53	89	62.7%
Overall	431	144	287	66.6%
